# Bird embryos uncover homology and evolution of the dinosaur ankle

**DOI:** 10.1038/ncomms9902

**Published:** 2015-11-13

**Authors:** Luis Ossa-Fuentes, Jorge Mpodozis, Alexander O Vargas

**Affiliations:** 1 Departamento de Biología, Laboratorio de Ontogenia y Filogenia, Facultad de Ciencias, Universidad de Chile, Las Palmeras 3425, Ñuñoa, Santiago 7800003, Chile; 2 Departamento de Biología, Laboratorio de Neurobiología y Biología del Conocer, Facultad de Ciencias, Universidad de Chile, Las Palmeras 3425, Ñuñoa, Santiago 7800003, Chile

## Abstract

The anklebone (astragalus) of dinosaurs presents a characteristic upward projection, the ‘ascending process' (ASC). The ASC is present in modern birds, but develops a separate ossification centre, and projects from the calcaneum in most species. These differences have been argued to make it non-comparable to dinosaurs. We studied ASC development in six different orders of birds using traditional techniques and spin–disc microscopy for whole-mount immunofluorescence. Unexpectedly, we found the ASC derives from the embryonic intermedium, an ancient element of the tetrapod ankle. In some birds it comes in contact with the astragalus, and, in others, with the calcaneum. The fact that the intermedium fails to fuse early with the tibiale and develops an ossification centre is unlike any other amniotes, yet resembles basal, amphibian-grade tetrapods. The ASC originated in early dinosaurs along changes to upright posture and locomotion, revealing an intriguing combination of functional innovation and reversion in its evolution.

## Abstract (web summary)

The anklebone of dinosaurs presents the ‘ascending process' (ASC), a projection also found in modern birds, yet the ASC in birds has unique developmental characteristics. Here, the authors show that the ASC in six birds develops from an ancient element of the tetrapod ankle in a way that resembles basal tetrapods.

## Graphical abstract

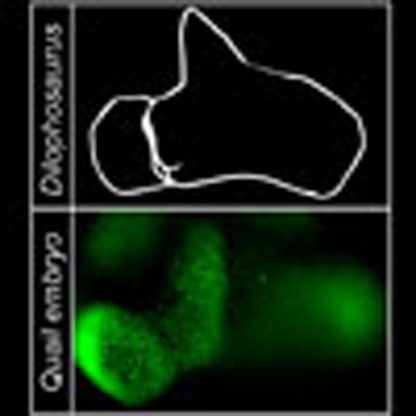

Modern amniotes typically develop two ossification centres in their proximal (upper) ankle: the astragalus (anklebone) and the calcaneum (heel bone, also named fibulare)^1^,^2^. The astragalus of theropod dinosaurs presents a triangular ascending process (ASC) projecting on the anterior surface of the tibia (shank bone)^3^. Early evolutionists T. Huxley and C. Gegenbaur noted that an ASC is also present in embryonic and juvenile birds, as a part of their case for dinosaur–bird relatedness^4^,^5^. The ASC of birds develops a third proximal ossification centre^6^,^7^ that is absent in other amniotes^1^,^8^, even in crocodilians, bird's closest living relatives^9^. The early cartilaginous development of this third ossification is controversial. E. Morse, another early evolutionist, used drawings to report that this ossification could be traced back to an early embryonic cartilage, the intermedium^10^. This would be remarkable. In non-avian amniotes (including crocodilians) the intermedium fuses early with another early cartilage, the tibiale, to conform a single astragalus cartilage^11^,^12^. An intermedium that remains separate and develops its own ossification centre is otherwise only known in amphibians^1^,^2^,^11^. Morse's account was disregarded by more recent studies. Published photographs do not document the presence of an intermedium in birds^13^,^14^,^15^, and only two early proximal cartilages are acknowledged: the tibiale (considered the sole precursor and equivalent of the astragalus) and the calcaneum^13^,^14^. Modern studies report that the ASC originates as a cartilaginous projection of the astragalus^14^ or, alternatively, from a late-forming, neomorphic ‘pretibial' cartilage^16^. Current photographic documentation is inadequate to assess these divergent accounts. We therefore sought to obtain detailed information on embryonic structure from six different orders of birds, including the chilean tinamou, a member of the anciently diverged Paleognathae. We used traditional techniques for cartilage staining in whole mounts and stacks of histological sections, and studied quail with a new technique for whole-mount immunofluorescence of embryonic cartilages^17^. Traditional techniques for three-dimensional (3D) visualization do not allow antibodies to penetrate whole cartilages, and do not maximize tissue transparency (‘invisibility'). Here we report that the embryonic intermedium is present, fails to fuse with the tibiale and becomes the ASC. This reveals an intriguing evolutionary change in this specialized dinosaurian trait, which acquired amphibian-like development. The actual modularity of the ASC also helps to explain its variable position in the evolution of modern birds, projecting from the calcaneum in Neognathae, rather than the astragalus.

## Results

### The intermedium is present in the embryonic ankle of birds

**Figure 1:**
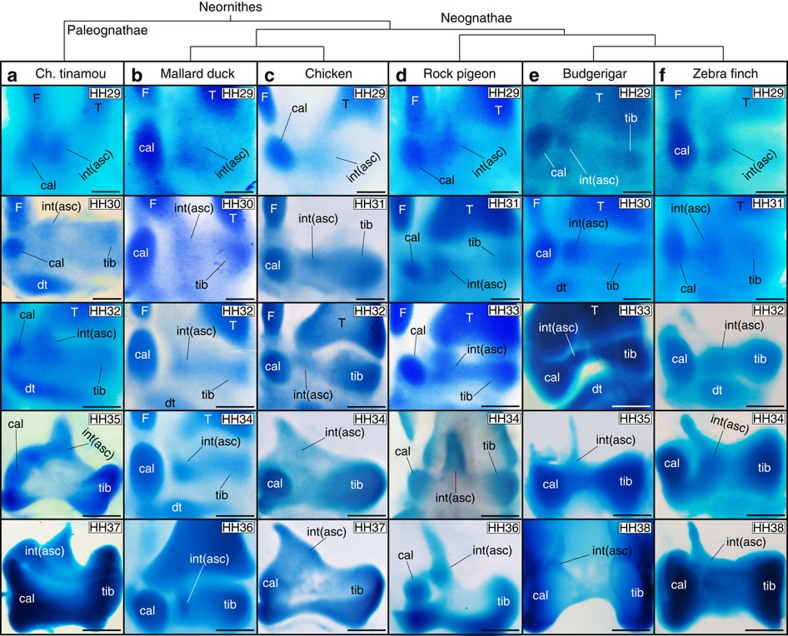
The intermedium is present in birds and it becomes the ascending process. Whole-mount cartilage staining is shown for (**a**) Chilean tinamou, (**b**) mallard duck, (**c**) chicken, (**d**) rock pigeon, (**e**) budgerigar and (**f**) zebra finch. At HH29-HH31 three condensations appear, from lateral to medial: the calcaneum (also known as fibulare), intermedium and tibiale. Following the intermedium into later stages (HH32-35) reveals that it becomes the ascending process. At late stages (HH36), the three proximal cartilages of the ankle fuse. Five embryos per stage were used for Chilean Tinamou, Mallard Duck and Rock Pigeon. Ten embryos per stage were used for Zebrafinch, Budgerigar and Chicken. asc, ascending process cal, calcaneum; dt, distal tarsals; F, fibula; int, intermedium; T, tibia; tib, tibiale. Scale bars, Stages HH29-31, 100 μm; HH34, 200 μm; HH36-38, 300 μm.

**Figure 2:**
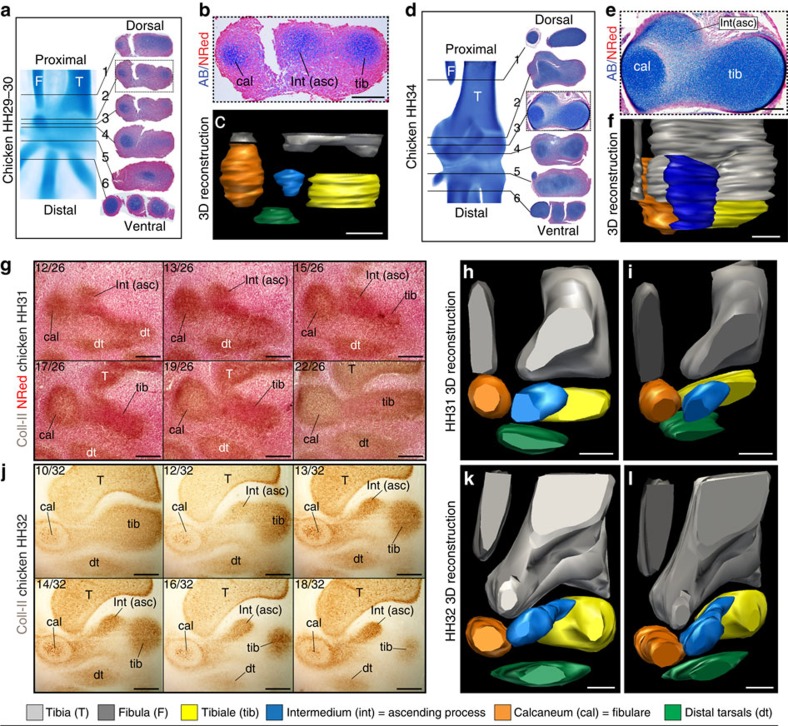
Histological sections confirm persistent modularity of the intermedium. (**a**) Select sections in a chicken hindlimb stage HH29-30. (**b**) Zoom-in of the fibulare, intermedium and tibiale. (**c**) 3D reconstruction from the complete HH29-30 stack (thickness=10 μm). (**d**) Selected sections at stage HH34. (**e**) Zoom-in shows the intermedium on the dorsal surface of the tibia, remaining separate from the fibulare and tibiale. (**f**) 3D reconstruction from the complete HH34 stack (thickness=12 μm). The thin lateral rod reaching down to the calcaneum is connective tissue left behind after distal fibular reduction. (**g**,**j**) Stacks of dorsoplantar histological sections in an immunohistochemistry assay against Collagen type-II in chicken hindlimbs stage HH31 and HH32, respectively, revealing three separate condensations corresponding to the calcaneum, intermedium and tibiale (see sections 12/26 to 22/26 for HH31 (**g**) and 13/32 to 18/32 for HH32 (**j**)). (**h**,**i**) 3D reconstruction from the complete HH31 stack (thickness=10 μm) showing dorsal (**h**) and lateral (**i**) views. (**k**,**l**) 3D reconstruction from the complete HH32 stack (thickness=10 μm) showing dorsal (**k**) and lateral (**l**) views. Five embryos of *Gallus gallus* per stage were used. asc, ascending process; cal, calcaneum; dt, distal tarsals; F, fibula; int, intermedium; T, tibia; tib, tibiale. Scale bars (**a**–**f**), 100 μm; (**g**–**l**), 150 μm.

The cartilaginous development of the bird ankle is shown in Fig. 1, which can be compared with the common pattern for non-avian amniotes in Supplementary Fig. 1. While other studies have failed to document an intermedium in birds, we found that it was present in all six orders examined. It forms around stage HH29, in the usual pattern for amniotes: after the calcaneum and before the tibiale, temporally as well as spatially (from lateral to medial, Fig. 1, HH29-31. Compare with Supplementary Fig. 1a,b). The tibiale has been labelled alternatively as a centrale in other amniotes^11^,^12^; however, we use the term tibiale following previous embryological studies of birds. Diffuse alcian blue staining is observed between elements, connecting the intermedium and tibiale, which may explain why modern studies only report a large tibiale^13^. However, distinct centres of cartilage formation for the tibiale and intermedium are evident (Fig. 1), especially on dissection (Supplementary Figs 2–4) and in histological sections (Fig. 2 and Supplementary Figs 5 and 6a). The latter reveals that ‘diffuse cartilage' corresponds to weak and disperse staining in the disorganized mesenchyme between elements, which is also present between the tibiale and calcaneum (Supplementary Fig. 6b).

### The intermedium becomes the ASC of birds

**Figure 3:**
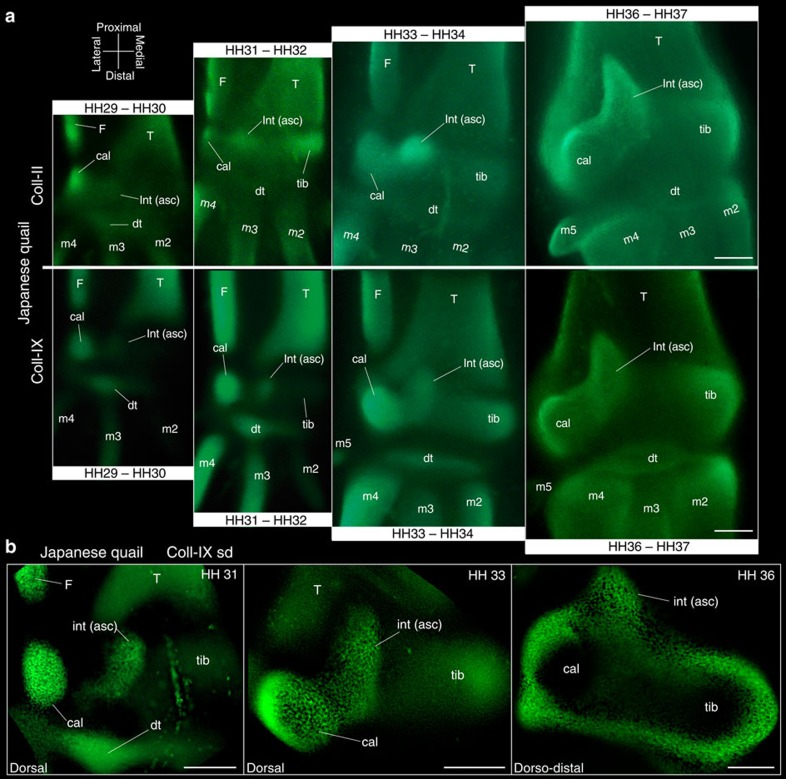
Expression of Collagen type-II and Collagen type-IX in the embryonic ankle of the Japanese quail. (**a**) Developmental series showing the three separate proximal cartilages in the early embryonic ankle, and continuity of the intermedium (HH31) with the ascending process. (**b**) Developmental series of Collagen type-IX expression as observed using spin-disc confocal microscopy. Five embryos of *Coturnix japonica* for each stage were used for immunofluorescence of each primary antibody. asc, ascending process cal, calcaneum; dt, distal tarsals; F, fibula; int, intermedium; m, metatarsus; T, tibia; tib, tibiale. Scale bars, 100 μm.

In non-avian amniotes, the intermedium fuses completely with the tibiale, forming a single large astragalus cartilage (see wreath lizard in Supplementary Fig. 1b). In contrast, the intermedium of birds does not fuse to the tibiale. Rather, it acquires an elongate shape and extends on the anterior face of the distal tibia, becoming the ASC (stage HH34 in Fig. 1 and Supplementary Figs 2–4). At later stages, all three proximal cartilages fuse into a single large cartilage that forms a ‘distal cap' to the tibia (Fig. 1, HH36). Three proximal ossifications develop thereafter within this cap, at positions corresponding to each early cartilage (Supplementary Figs 7 and 8). Long after hatching, these ossifications fuse to each other, and then fuse to the tibia, producing the adult tibiotarsus^14^. In contrast, in non-avian amniotes, only two proximal ossification centres are formed, corresponding to the astragalus and calcaneum (Supplementary Fig. 8). Traditional alcian blue staining binds to polysaccharides that are highly concentrated in cartilage but are also produced in other connective tissues^18^. Collagen type-II (*Coll II*) is only expressed in cartilage and has not been reported elsewhere^19^,^20^. In histological sections of chicken, *Coll II* confirms a distinct centre of cartilage formation for the intermedium (Fig. 2g–l). Unlike whole mounts, histological sections provide information on tissue organization and aspects hidden by element superposition. However, separate elements and complex shapes are hard to tell apart using two-dimensional (2D) sections. 3D surface reconstruction software from stacks can help overcome this problem (Fig. 2c,f,h–l and Supplementary Fig. 9); however, slice thickness limits resolution, and researchers must manually trace the limits of elements. In turn, whole-mount immunostaining allows the use of spinning disc microscopy at high, single-cell resolution for optical sectioning and 3D reconstruction^17^. We used whole mounts to observe the expression of both *Coll II* and Collagen type-IX (*Coll IX*). *Coll IX* relates to early cartilage differentiation: it is expressed shortly after cartilage formation, but before hypertrophy^20^. Both *Coll II* and *Coll IX* show the presence of an independent intermedium that thereafter becomes the ASC (Fig. 3 and Supplementary Movies 1–3).

## Discussion

The ASC once played an important role in the debate over the origin of birds. While the ASC of dinosaurs was considered a projection of the astragalus, the ASC in birds was argued to be a separate ‘pretibial bone', developing from a neomorphic cartilage^16^. It was also pointed out that in neognathous birds, the ASC projects from the calcaneum rather than the astragalus as in dinosaurs^6^,^21^ (see neognathous quail Fig. 3a). The ASC projects from the astragalus in paleognaths^14^ (see tinamou in Supplementary Fig. 10b); however, this was argued to be a derived condition among birds^22^. Morphological similarity of the ASC in birds and dinosaurs was proposed to reflect convergence, not homology. If so, the ASC could not be used as support for dinosaur–bird relatedness^6^,^16^,^21^. Currently, the origin of birds from dinosaurs has been settled through modern techniques of phylogenetic analysis and an accumulation of evidence, including intermediate fossil taxa, feathered dinosaurs (reviewed in ref. 23) and molecular phylogenies using collagen recovered from fossils^24^. The ASC of birds and non-avian dinosaurs is now considered homologous^25^, being one of their numerous shared-derived traits (synapomorphies). The ASC has remained morphologically similar and phylogenetically continuous from basal forms such as *Dilophosaurus,* to *Archaeopteryx* and modern birds^26^,^27^. Furthermore, any developmental differences between the ASC of birds and dinosaurs are insufficient evidence for convergence^14^. The phylogenetically correct inference is that they represent evolutionary variations in the development of a homologous character (a well-documented phenomenon^28^,^29^).

Our new data on early development show how the ASC is actually not a projection of either astragalus or calcaneum, but an independent element—the intermedium. The actual modularity of the ASC helps to explain its labile position in the evolution of different bird lineages, coming closer to the astragalus in paleognathous birds, and closer to the calcaneum in neognathous birds^14^. We have also conclusively discarded the possibility that the ASC derives from a neomorphic cartilage. Birds present the same highly conserved developmental pattern as all known amniotes that allow easy identification of the intermedium, which forms after the calcaneum (fibulare) but before the tibiale, both temporally and spatially (from lateral to medial)^11^. Our numerous developmental series of birds demonstrate that this ancient element of the tetrapod ankle is in fact the embryological precursor of the avian ASC.

Importantly, our discovery uncovers an evolutionary change in birds that is unprecedented among amniotes: the decoupling of the intermedium from the astragalus to develop separately and form an ossification, a condition resembling remote amphibian-grade ancestors (Supplementary Fig. 11). This cannot be described as a mere delay in its incorporation to the astragalus: the intermedium remains separate until late fusion of all proximal elements, including the calcaneum, into the large ‘distal cap' to the tibia. This distal cap includes the calcaneum and is non-equivalent to the astragalus. Rather, it is part of the late fusion events leading to the composite tibiotarsus of birds. A proper astragalus is never formed in birds, although the ossification that develops in the tibiale is arguably homologous to that in the astragalus of other amniotes. Beyond this nomenclatural challenge, we may ask about the mechanisms underlying the evolutionary decoupling of the astragalus. BMP-Smad4 and Wnt signalling are involved in the early formation of skeletal condensations^30^ and maintenance of the interzone that stops elements such as phalanges from fusing to each other 31. These molecular pathways are also involved in the patterning of other highly individualized structures^32^. Their local regulation may prevent the early intermedium from fusing to the tibiale, thus recovering its individuality. However, the mechanisms that pattern the ankle region are poorly understood, and much work remains to assess if molecular patterning is amphibian-like in the ankle of birds.

The decoupling of the ASC from the astragalus raises interesting questions about the evolutionary circumstances in which it occurred. Early morphological specialization of part of the astragalus into a small, pyramid-like ASC is well documented by taxa such as *Lagosuchus* and early dinosaurs (Supplementary Fig. 12a)^33^,^34^. In theropods such as *Dilophosaurus*, it became larger, flat and triangular, as in birds^3^. An intriguing possibility is that the ASC first evolved as a bony projection of the astragalus before becoming a separate ossification. Alternatively, the ASC may have originated as a separate ossification from the start. In modern birds, the three proximal ossifications remain separate long after hatching (see 14 days post hatching in chicken, Supplementary Fig. 7) and only fuse when approaching maturity (40 day post hatching in chicken^14^), leaving no suture lines in the adult. Fossils of skeletally immature dinosaurs can be informative; however, little information is available on ankle development. A suture line for a separate ossification in the ASC has been reported for *Fruitadens*, which, within dinosaurs, is maximally distant from birds phylogenetically along with other ornithischians dinosaurs^35^.

This suggests that an ASC ossification could have been present from the very origin of dinosaurs, but too much data are missing from taxa in intermediate phylogenetic positions. Thus, convergence (homoplasy) cannot be discarded (Supplementary Fig. 12a). In taxa closer to birds, an alleged suture line between the ASC and the main body of the astragalus has been reported for the basal theropod *Dilophosaurus*^36^. However, similar lines are observable across the specimen, and all seem to be fractures (Supplementary Movie 4). It is likely that the specimen is an adult and thus would not show any sutures. Inner spaces of spongy bone tissue could indicate previously separate ossifications^37^, but this requires examination of internal structure. CT scans and more specimens of skeletally immature dinosaurs may help to clarify the early evolution of the ASC. As for basal birds, new data are becoming available from juvenile enantiornithines from the Lower Cretaceous of China. An unnamed specimen reveals a tripartite structure, poorly ossified at the centre (Supplementary Fig. 12b–d) resembling a distal tibial cap with three ossification centres (compare with Supplementary Fig. 10a). Another unnamed specimen at a more advanced developmental stage shows an ASC that is clearly detached from the astragalus and calcaneum (Supplementary Fig. 12e,f). This suggests that a separate ASC ossification was already present in this early lineage of toothed birds.

The ASC first appeared in the ‘advanced mesotarsal' ankle joint of the Ornithodira (dinosaurs and their close relatives). In this type of joint, the proximal elements become fixed to the lower leg, and movement occurs only at their distal articular surface. The ASC arguably braces the astragalus against the lower leg^38^. This joint evolved along functional innovations in upright locomotion and posture^37^. The ASC became larger and more bird-like in theropod dinosaurs, which specialized further in bipedal running^39^. In contrast with these new functions, the embryology of the ASC uncovers the amphibian-like development of this trait. In many cases of evolutionary reversion, lost traits are seldom regained in the same form and can easily be mistaken for entirely new structures^40^. Often, only ontogeny will uncover an ancient developmental pathway^41^. The evolution of the ASC may be especially insightful about the relation between adaptive innovation and reversion.

The early formation of the limb skeleton is often assumed to be well known from ‘classic' published lines of work using traditional techniques and model species. However, it is worth revising this basic information. New methods and better taxon sampling can provide increased detail and substantial revision, with important evolutionary implications. In addition to the fossil record, reliable embryological data from birds are important to understand their evolution from dinosaurs^17^.

## Methods

### Animal embryos

All procedures were formally approved by the Comité de Etica de la Facultad de Ciencias, Universidad de Chile, which certifies compliance with all aspects required for government funding (http://www.conicyt.cl/fondecyt/2012/10/31/bioetica/). None of the wild species used is in a conservation category of concern (http://www.iucnredlist.org). *Octodon degus* fetus and newborn were obtained from gravid females bred in captivity and maintained in an institutional animal facility. Eggs from *Lioalemus lemniscatus* (wreath lizard) were laid by gravid females captured with field permits of the Servicio Agrícola y Ganadero (SAG, Government of Chile) and were incubated following published procedures for *L. tenuis*^42^. Fertilized eggs of *Gallus gallus* (Chicken, Galliformes), *Anas platyrhynchos* (Mallard duck, Anseriformes)^43^ and *Nothoprocta perdicaria* (Chilean tinamou, Tinamiformes) were purchased from local farms: Chorombo S.A., Avícola Metrenco and Tinamou Chile (perdiz.cl). Fertilized eggs of *Columba livia* (Rock pigeon, Columbiformes), *Taeniopygia guttata* (Zebra finch, Passeriformes) and *Melopsittacus undulatus* (Budgerigar, Psittaciformes) were obtained from birds kept at facilities of the Faculty of Science, University of Chile. Fertilized eggs of *Vanellus chilensis* (Chilean lapwing, Charadriiformes) were collected with permission from SAG (DS-P and DN-L). Photographs of a 65-day *Alligator mississippiensis* embryo kept at the Field Museum of Natural History (FMNH 250664) were kindly provided by Alan Resetar.

### Cartilage and bone staining

Embryos were fixed in 100% methanol for 2–3 days at room temperature (RT). Methanol was replaced by 5:1 ethanol/acetic acid solution with 0.03% 8G alcian blue for 2 days at RT in an orbital shaker. Late embryos for bone staining were submerged in 0.03% alizarin red: 0.5% KOH solution for 1–4 h at RT and washed in distilled water. Then, embryos were cleared in a sequence of 1:3, 1:1 and 3:1 glycerol/water, and photographed in a stereoscopic microscope.

### Serial histological sections and immunohistochemistry

Five embryos of *G. gallus* per stage were fixed in 4% paraformaldehyde for 2 h at RT or overnight at 4 °C. Then, hindlimbs were dissected, washed in PBS 1% and dehydrated in ethanol increasing concentrations (50% to absolute ethanol). Limbs were cleared in Neoclear and vacuum-embedded in Paraplast for 1 h. The paraplast-embedded material was cut into 10–12-μm-thick transversal sections, rehydrated and stained with alcian blue/nuclear red. 3D surface reconstruction of embryonic cartilages from stacks of histological sections was made using the 3D visualization module of the Neurolucida 9.1 software. For immunochemistry essays against *Coll II*, five embryos of *G. gallus* for stage HH32 were used. Using the same procedure described above, 10-μm-thick dorso-palmar sections were obtained. Then, the sections were bleached in Dent's bleaching (4:1:1 methanol:DMSO:H_2_O_2_) for 24 h at RT. Antigen retrieval was carried out by heat-induced sodium citrate solution (10 mM, pH=6.0) for 30 min at 80 °C. Then, the sections were digested with 2 mg ml^−l^ of hyaluronidase (Sigma) in PBS for 2 h at 37 °C. Sections were rehydrated in PBS 1% triton (PBST) and incubated in primary antibodies overnight at 4 °C in an orbital shaker. The primary antibody was diluted in 2% horse serum and 5% dimethylsulphoxide (DMSO) in PBST at the following concentrations: 1:40 *Coll II* (II-II6B3, DSHB). Secondary antibody anti-mouse made in donkey coupled to horseradish peroxidase (715-035-150, Jackson ImmunoResearch) were diluted 1:300 in PBST, 2% horse serum and 5% DMSO. Sections were kept in secondary antibody for 2 h at 4 °C in an orbital shaker. They were washed again in PBST six times for 1 h. Sections labelled with horseradish peroxidase were revealed with diaminobenzidine substrate (11718096001, Roche Applied Bioscience).

### Whole-mount immunofluorescence

Embryos were fixed in Dent's Fix (4:1 methanol/DMSO) for 2 h at RT, dehydrated in 100% methanol and left at −80 °C overnight. Before immunostaining, they were bleached in Dent's Bleaching (4:1:1 methanol/DMSO/H_2_O_2_) for 24 h at RT. For anti-collagen immunostaining, embryos were fixed and bleached as above. Then, hindlimbs were dissected and digested with 2 mg ml^−l^ of hyaluronidase (Sigma) in PBS for 2 h at 37 °C. Embryos were rehydrated in PBST and incubated in primary antibodies for 2 days at 4 °C in an orbital shaker. Primary antibodies were diluted in 2% horse serum, 5% DMSO in PBST at the following concentrations: 1:40 *Coll II* (II-II6B3, DSHB) and 1:40 *Coll IX* (2C2-II, DSHB), and were washed in PBST (3 × 10 min and 3 × 1 h in an orbital shaker). Secondary antibodies anti-mouse (Alexa-488 Jackson ImmunoResearch, PA) were diluted in 5% goat serum and 5% DMSO in PBST and were incubated for 24 h at 4 °C. After that, they were washed, cleared with Urea 4 M and photographed in a fluorescent stereoscopic microscope (Nikon). For 3D reconstructions, 10-μm stacks were obtained in a spinning disk confocal microscope (Olympus) and projected in the cellSens software (analysis for Z stacks obtained for 3D reconstruction, 2D deconvolution/Nearest Neighbor analysis for removal of background fluorescence). This analysis avoids the effects of element superposition, which cannot be discarded in whole mounts using traditional alcian blue staining.

### Fossil specimens

Ankle materials of *D. wetherilli* specimen UCMP N° 37302 were directly examined by AOV at the collection of the Museum of Paleontology of the University of California, Berkeley. Jingmai O'Connor kindly provided original photographs of the hindlimbs of specimen STM34-1, an early juvenile enantiornithine from the Early Cretaceous Yixian Formation Liutiaogou Locality, Ningcheng, Chifeng City, Inner Mongolia (China) housed at the Tianyu Natural History Museum, Shandong, China, and photographs of another unnamed enantiornithine specimen IVPPV20289, also from the Early Cretaceous Yixian Formation, housed at the Institute of Vertebrate Paleontology and Paleoanthropology, Beijing, China. J. O'Connor assessed the interpretative drawings in Supplementary Fig. 12.

## Additional information

**How to cite this article:** Ossa-Fuentes, L. *et al.* Bird embryos uncover homology and evolution of the dinosaur ankle. *Nat. Commun.* 6:8902 doi: 10.1038/ncomms9902 (2015).

## Supplementary Material

Supplementary Figures and Supplementary References Supplementary Figures 1-12 and Supplementary References

Supplementary Movie 1 Whole mount Col9 immunostaining of Quail, stage HH29-30. Distinct domains of expression confirm the intermedium is present at stages HH29-30. 3D reconstruction of the embryonic ankle obtained from a spin-disc confocal microscope.

Supplementary Movie 2 Whole mount Col9 immunostaining of Quail, stage HH33. Distinct domains of expression confirm the ASC is derived from the intermedium. 3D reconstruction of the embryonic ankle obtained from a spin-disc confocal microscope.

Supplementary Movie 3 Whole mount Col9 immunostaining of Quail, stage HH36. At late stages, all proximal elements of the ankle coalesce into a single large cartilage that forms a "distal cap" to the tibia. 3D reconstruction of the embryonic ankle obtained from a spin-disc confocal microscope.

Supplementary Movie 4 Astragalus of the basal theropod Dilophosaurus. Alleged "suture lines" are actually indistinguishable from various fracture lines in the specimen. (video horizontally flipped to match orientation of other figures in this article)
