# Supplementary material for: Bird embryos uncover homology and evolution of the dinosaur ankle
Source: Nat Commun. 2015 Nov 13;6:8902. doi: 10.1038/ncomms9902 (PMC4660350; doi:10.1038/ncomms9902)
Supplement: Supplementary Figures and Supplementary References — Supplementary Figures 1-12 and Supplementary References [file ncomms9902-s1.pdf]

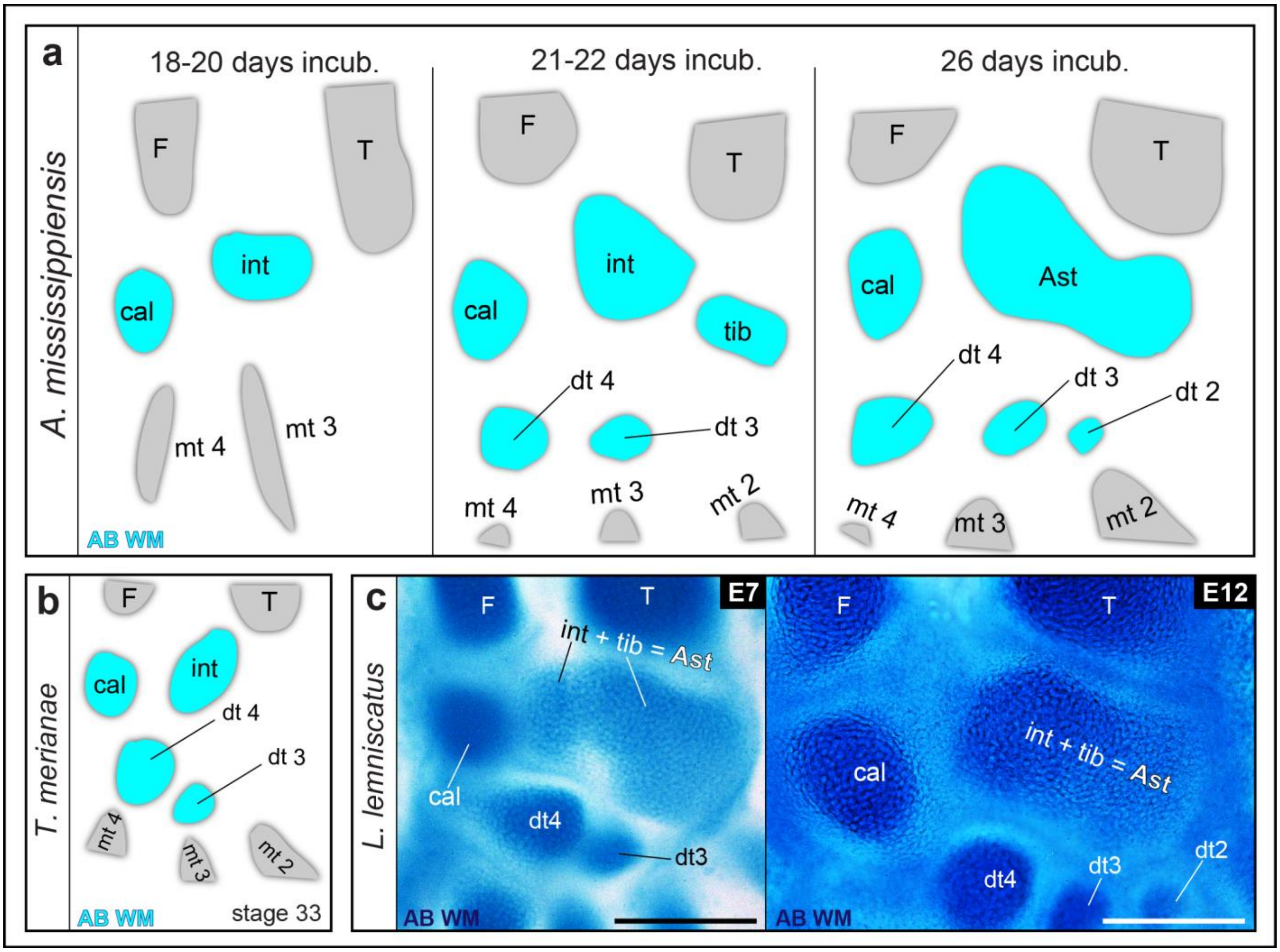

Supplementary Figure 1 | Cartilaginous stages in non-avian amniotes. (a) Drawing of early ankle development of *Alligator mississippiensis,* as reported by a previous study[1]. The intermedium is formed at 18-20 days, before the tibiale, which forms at 21-22 days, and then fuses to the intermedium, forming the astragalus **(b)** Early formation of the intermedium, before the tibiale, in the squamate *Tupinambis merinae* as reported in a previous study[2]. This pattern is common to all non-avian amniotes[3] **(c)** Fusion of the intermedium and tibiale in a squamate, the wreath lizard (*Lioalemus lemniscatus*). Intermedium and tibiale become closely appressed (E7-E10) and fuse completely into the composite astragalus cartilage, leaving no septum or mark (E12). In turtles, a third cartilage (a centrale) is reported to fuse thereafter to the astragalus[4]. 2 embryos per stage were used for wreath lizard. Abbreviations: T= tibia, F= fibula, int = intermedium, cal= calcaneum dt= distal tarsal, mt= metatarsal. Scale bars: 200 μm. All images are in dorsal (upper) view.

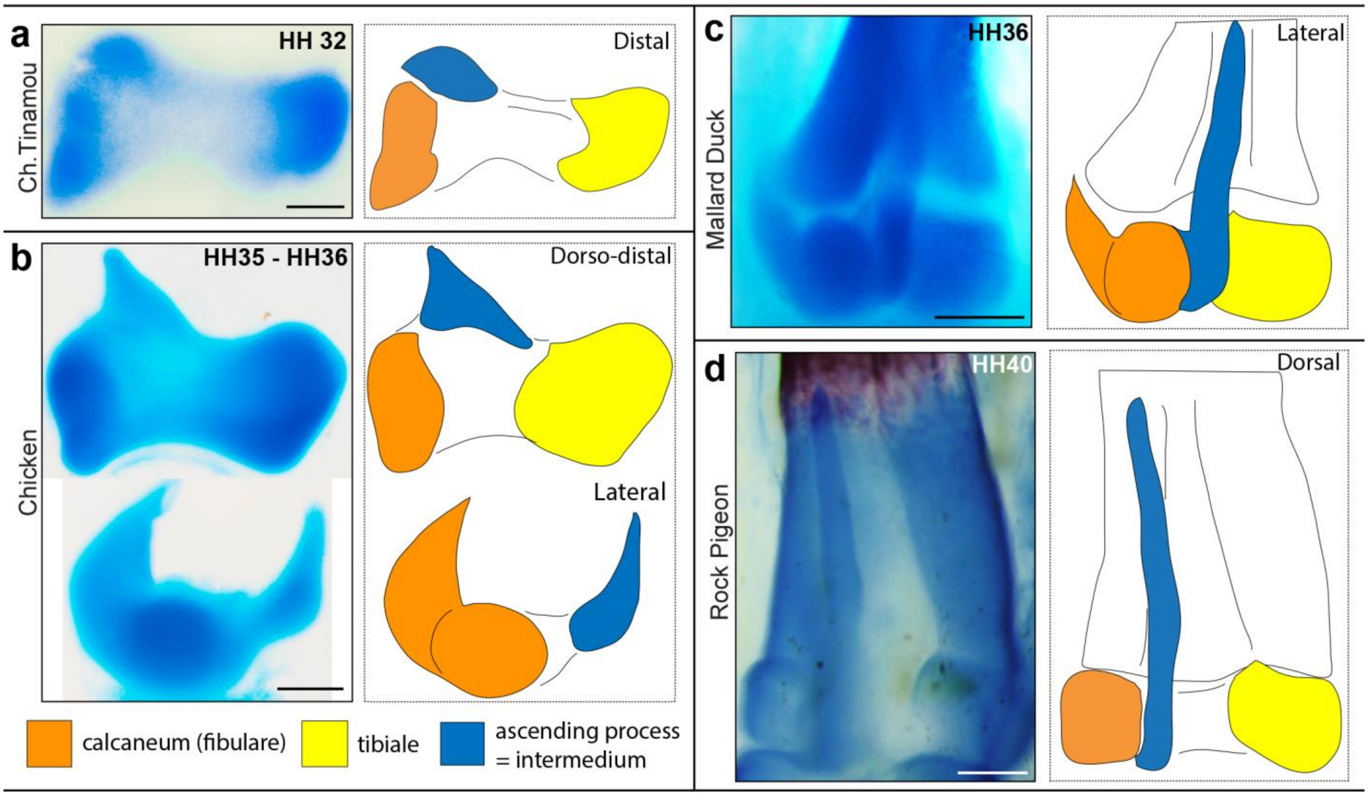

Supplementary Figure 2 | Proximal ankles of bird embryos showing a distinct centre of cartilage formation for the ASC (**a**) Dissected proximal elements of the Chilean tinamou in distal view (**b**) Dissected proximal elements of the chicken. Notice an upward process on the postero-lateral aspect of the calcaneum. Unlike the ASC, this upward process cannot be traced back to an early centre of cartilage formation, but forms as a late projection from the calcaneum (**c**) The ASC (= intermedium) at late stages of the mallard duck and (**d**) rock pigeon is an elongate, strut-like cartilage. 5 embryos per stage were used for Chilean Tinamou, Mallars Duck and Rock Pigeon. 10 or more embryos per stage were used for Chicken. Scale bars: (**a**) 100 μm, (**b**-**c**) 200 μm, (**d**) 300 μm.

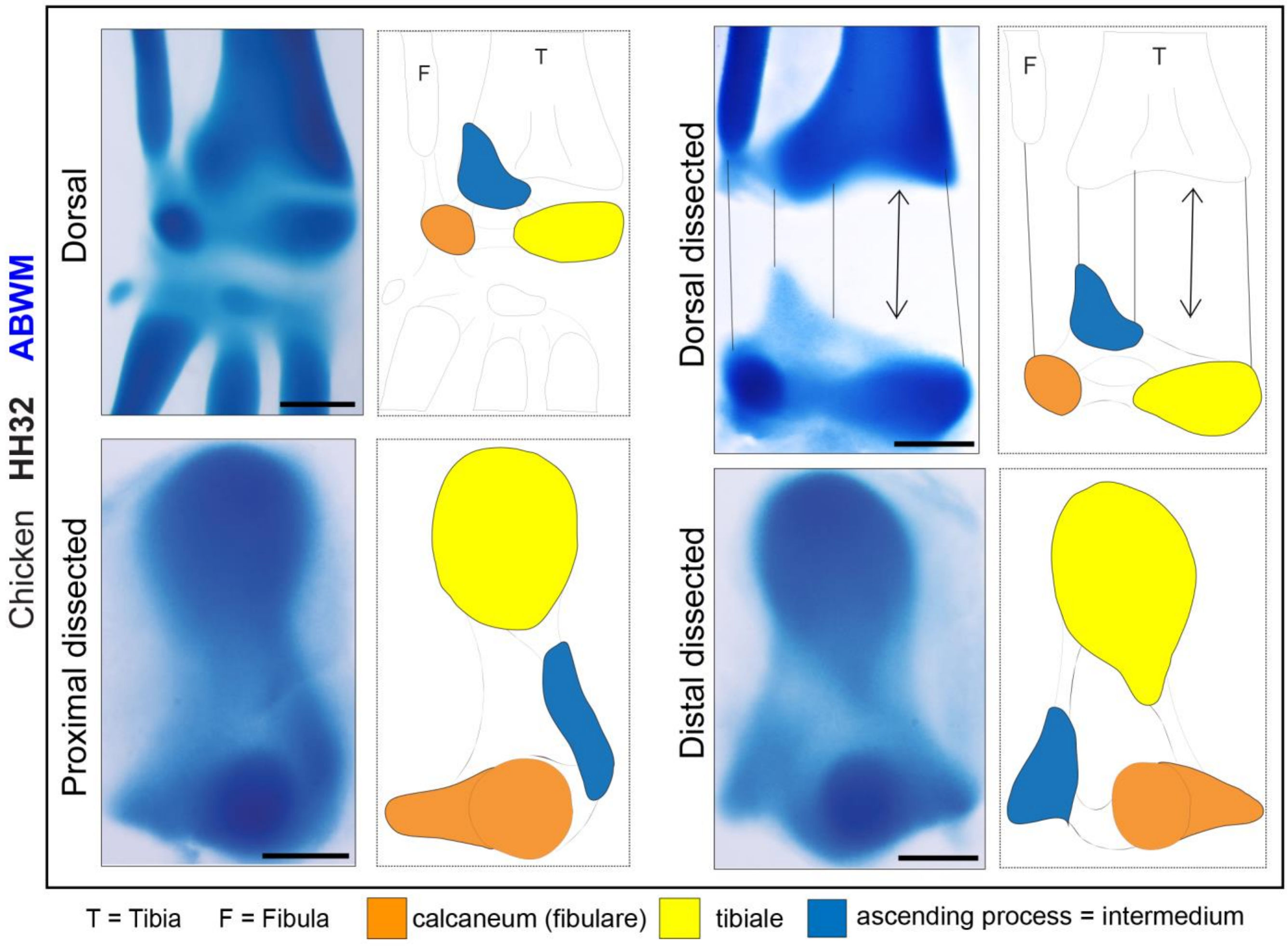

Supplementary Figure 3 | Dissection of the proximal ankle of a stage HH32 chicken embryo. Alcian Blue whole mounts for HH32. Without dissection, superposition of the intermedium with the antero-proximal tibia can make it hard to recognize as a distinct element. 10 embryos per stage were used for chicken. Scale bars: 100 µm.

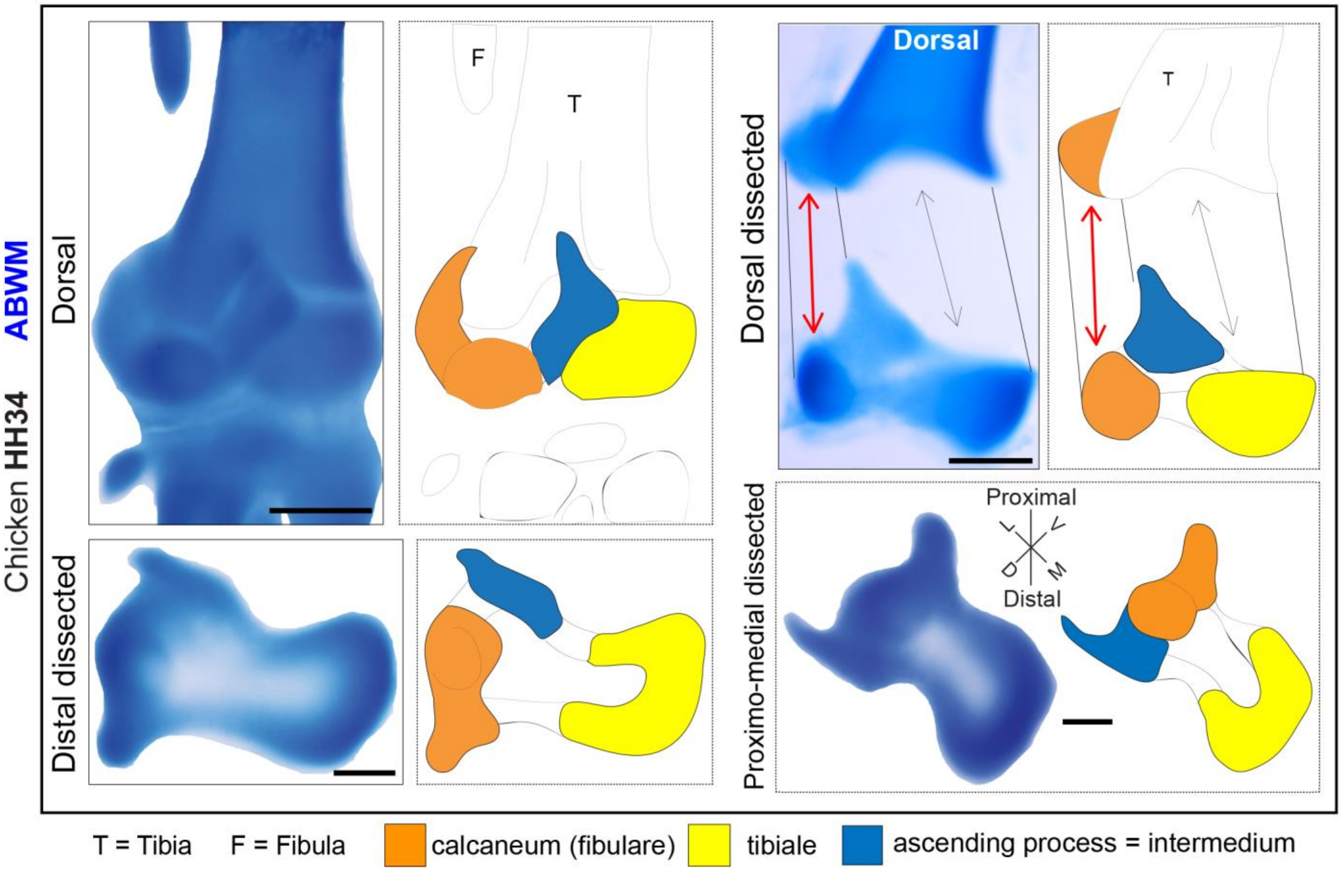

Supplementary Figure 4 | Dissection of the proximal ankle of a stage HH34 chicken embryo. Alcian Blue whole mounts for HH34. The red arrow indicates where the upward process of the postero-lateral calcaneum has broken off. 10 embryos per stage were used for chicken. Scale bars: 200 μm.

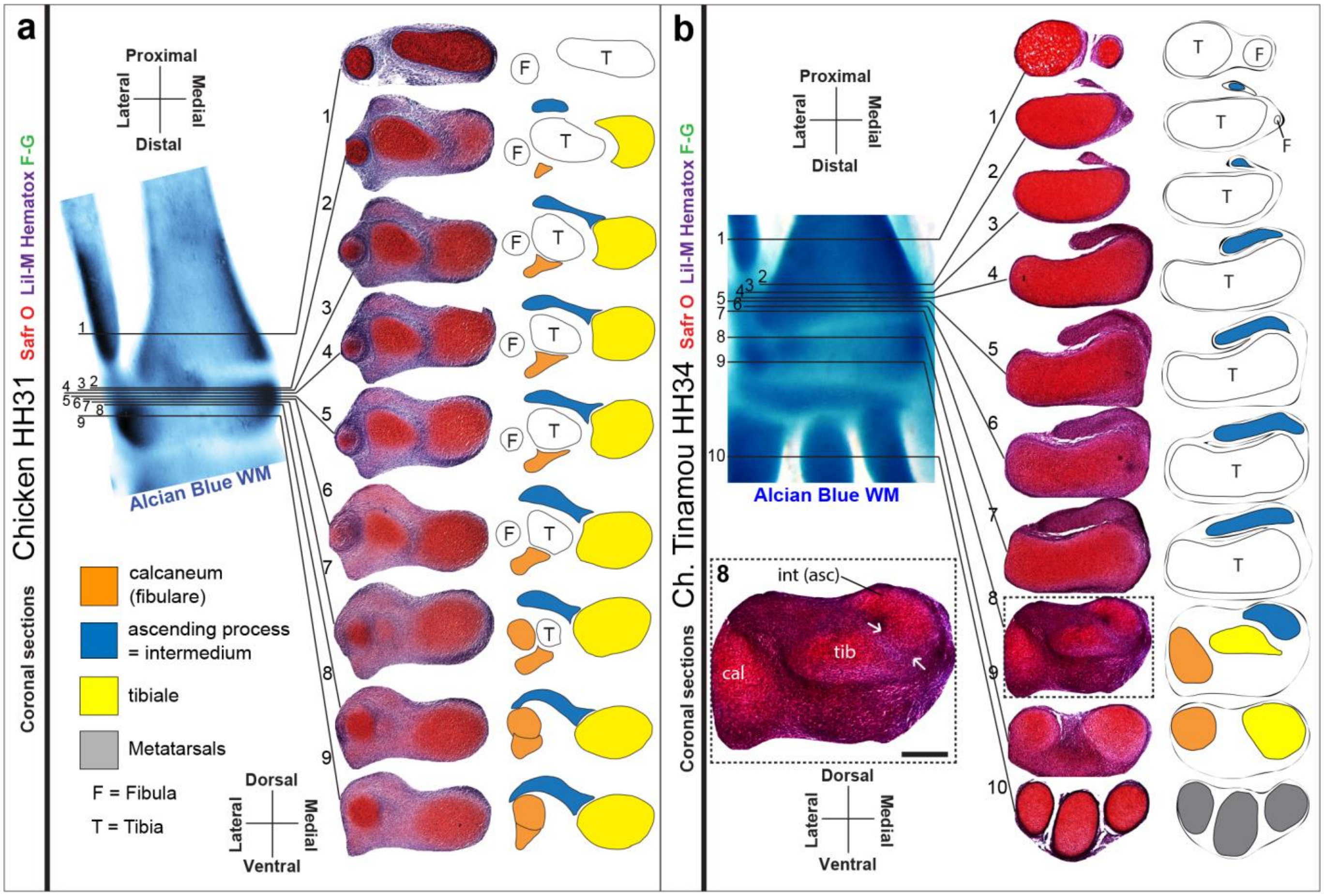

Supplementary Figure 5 | Histological sections of the proximal ankle in chicken and tinamou (**a**) HH31 chicken embryo confirms the ASC is a separate cartilage, rather than a projection of the astragalus. 5 embryos stage HH31 were used for chicken. (**b**) HH34 Chilean tinamou confirms the ASC is a separate cartilage. Despite proximity of the ASC with the tibiale, cartilage fusion has not occurred, remaining separate by distinct perichondrial limits (Arrows in the close-up of section 8 show, scale bar=200 μm.). 5 embryos stage HH 32 were used for Chilean tinamou. Sections were stained with Safranin-O for cartilage and Lilie-Mayer Hematoxilin/Fast green for nucleus/cytoplasm counterstain.

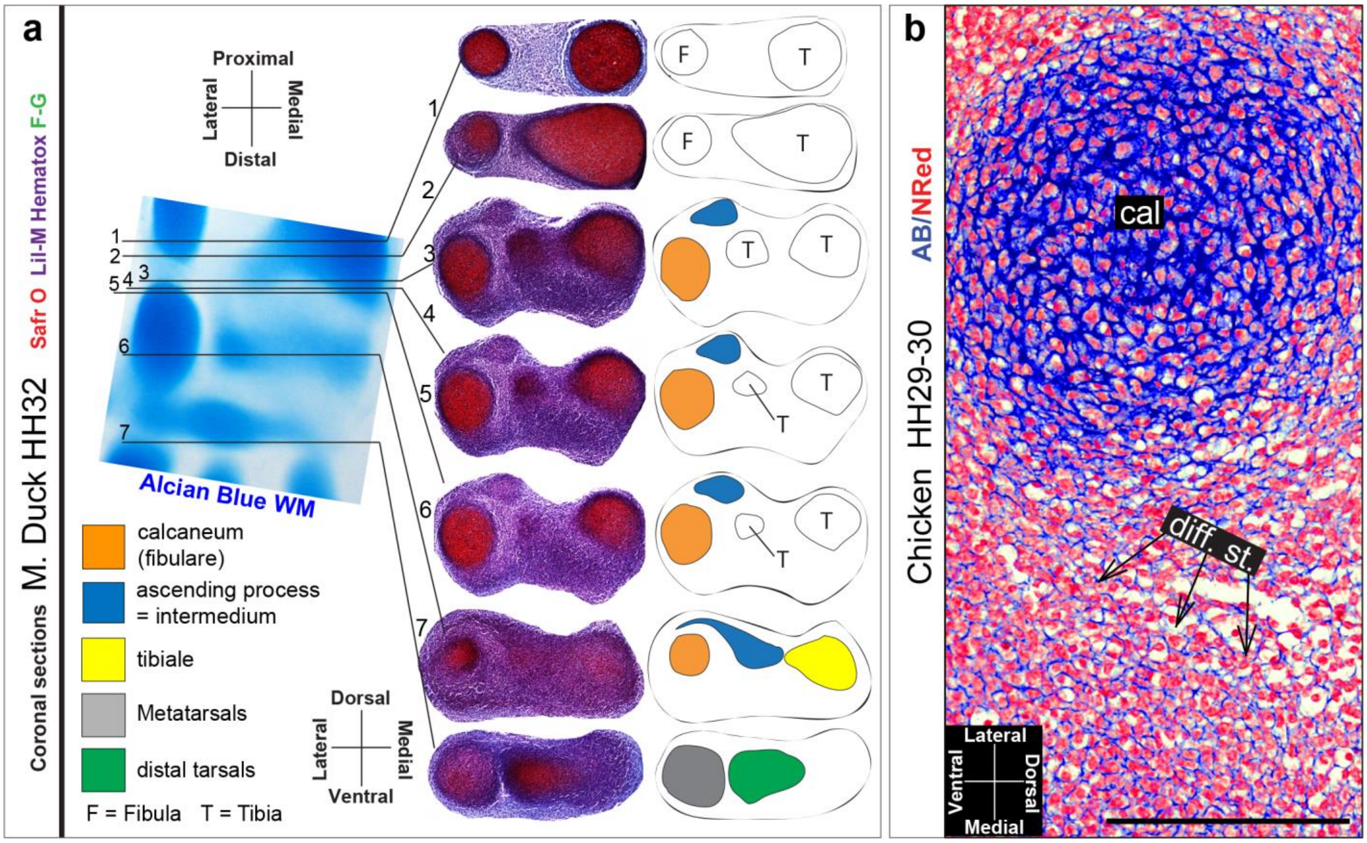

Supplementary Figure 6 | Histological sections of the proximal ankle in duck and chicken (**a**) Histological sections of HH32 mallard duck (stained with safranin-O for cartilage and Lilie-Mayer Hematoxilin/Fast green for nucleus/cytoplasm counterstain) confirm the ASC is a separate cartilage rather than a projection of the astragalus. 5 embryos stage HH32 were used for Mallard Duck. (**b**). **"**Diffuse cartilage" between ankle elements. Diffuse alcian blue staining that connects skeletal elements is often observed in whole mounts, which can lead to doubts on the actual number of elements and their limits. Histological sections reveal weak and diffuse alcian blue staining occurs (black arrows) in the disorganized tissue surrounding well-defined centres of cartilage formation. This picture shows diffuse staining between the calcaneum and intermedium. Scale bar =100 μm.

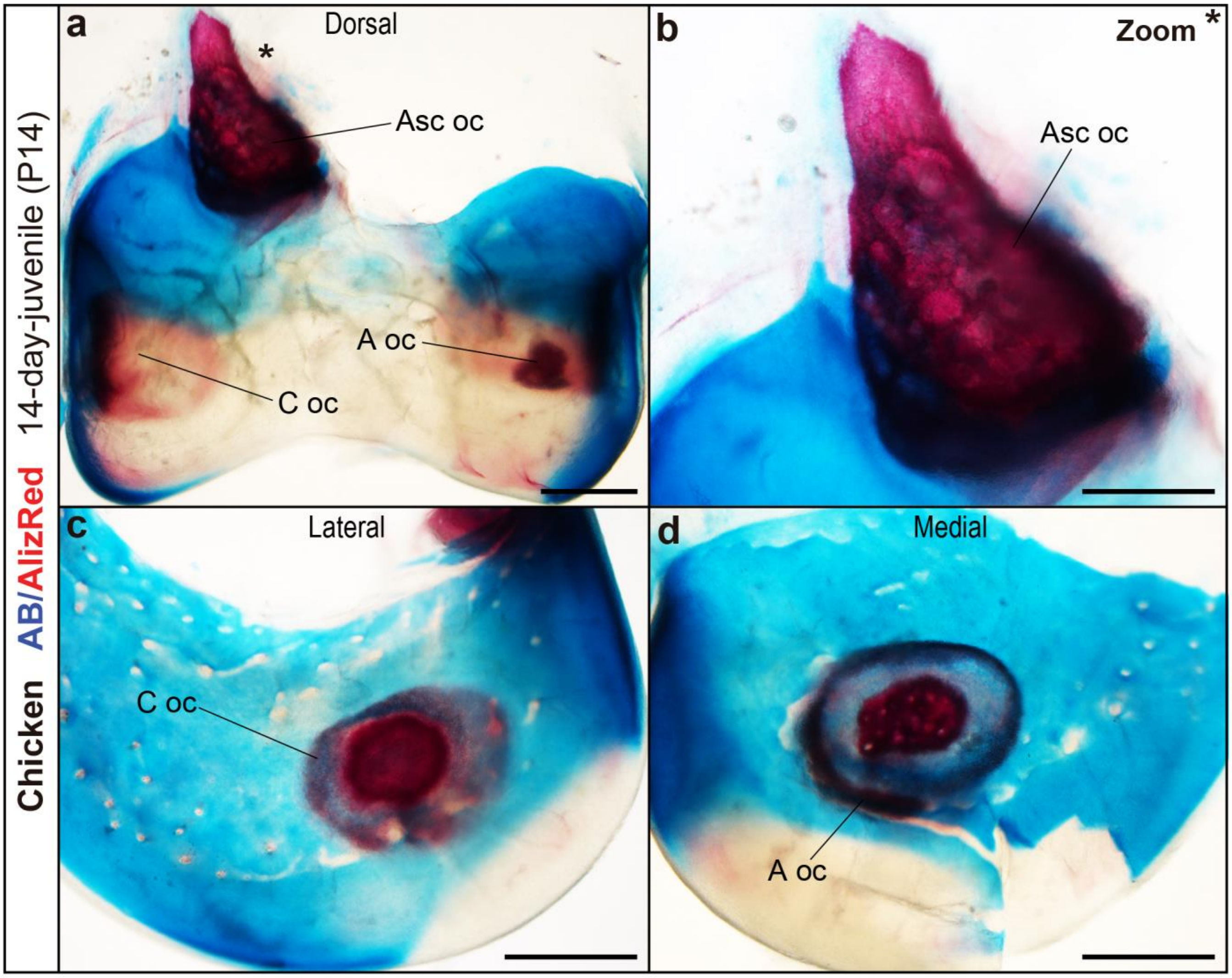

Supplementary Figure 7 | Post-hatching ossifications in the chicken ankle. a) At 14 days after hatching. The distal cap to the tibia shows three ossifications, still not fused to each other. In the adult, fusion of these ossifications is complete, leaving no suture lines. Abbreviations: C oc=Calcaneus ossification centre, A oc=Astragalus ossification centre, Asc oc=Ascending process ossification centre. Scale bars=1 mm.

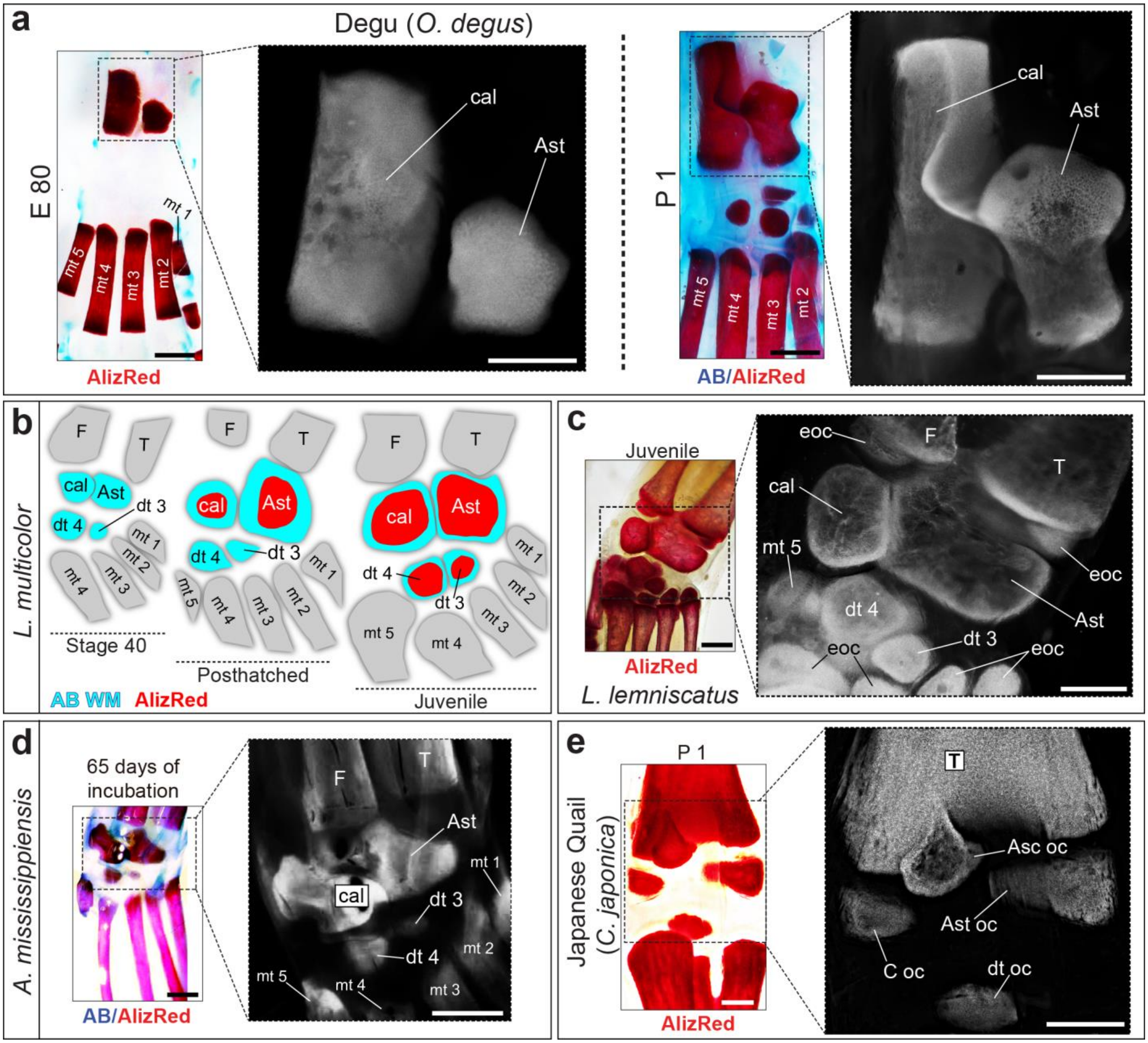

Supplementary Figure 8 | Proximal ossifications in amniote ankles (**a**) Fetus (E80) and newborn (P1) of degu (*Octodon degus*, Rodentia) shows the two proximal ossifications present in the ankle of crown amniotes, the calcaneum (lateral, left) and astragalus (medial, right). (**b**) Ankle development redrawn from a published study in the iguanid lizard *Liolaemus multicolor*[2] to show early ossifications of the astragalus and calcaneum (**c**) The ankle of a skeletally immature (juvenile) specimen of the wreath lizard *Lioalemus lemniscatus*. (**d**) 65 day embryo of *Alligator mississippiensis* (Crocodylia) showing two proximal ossifications (calcaneum and astragalus) (**e**) Newly hatched (P1) Japanese quail showing a third proximal ossification of birds, that of the ascending process (ASC), in addition to the astragalus and calcaneum. This ossification is an evolutionary innovation of birds, also known as the “pretibial bone”. 2 embryos per stage were used for Japanese quail, wreath lizard and degu.Abbreviations: T= tibia, C oc=Calcaneus ossification centre, A oc=Astragalus ossification centre, Asc=Ascending process, oc= ossification centre, dt oc=distal tarsals ossification centre. Scale bars: (**a**) 2 mm, (**c-d**) 2 mm, (**e**) 1 mm. All images are in dorsal (upper) view.

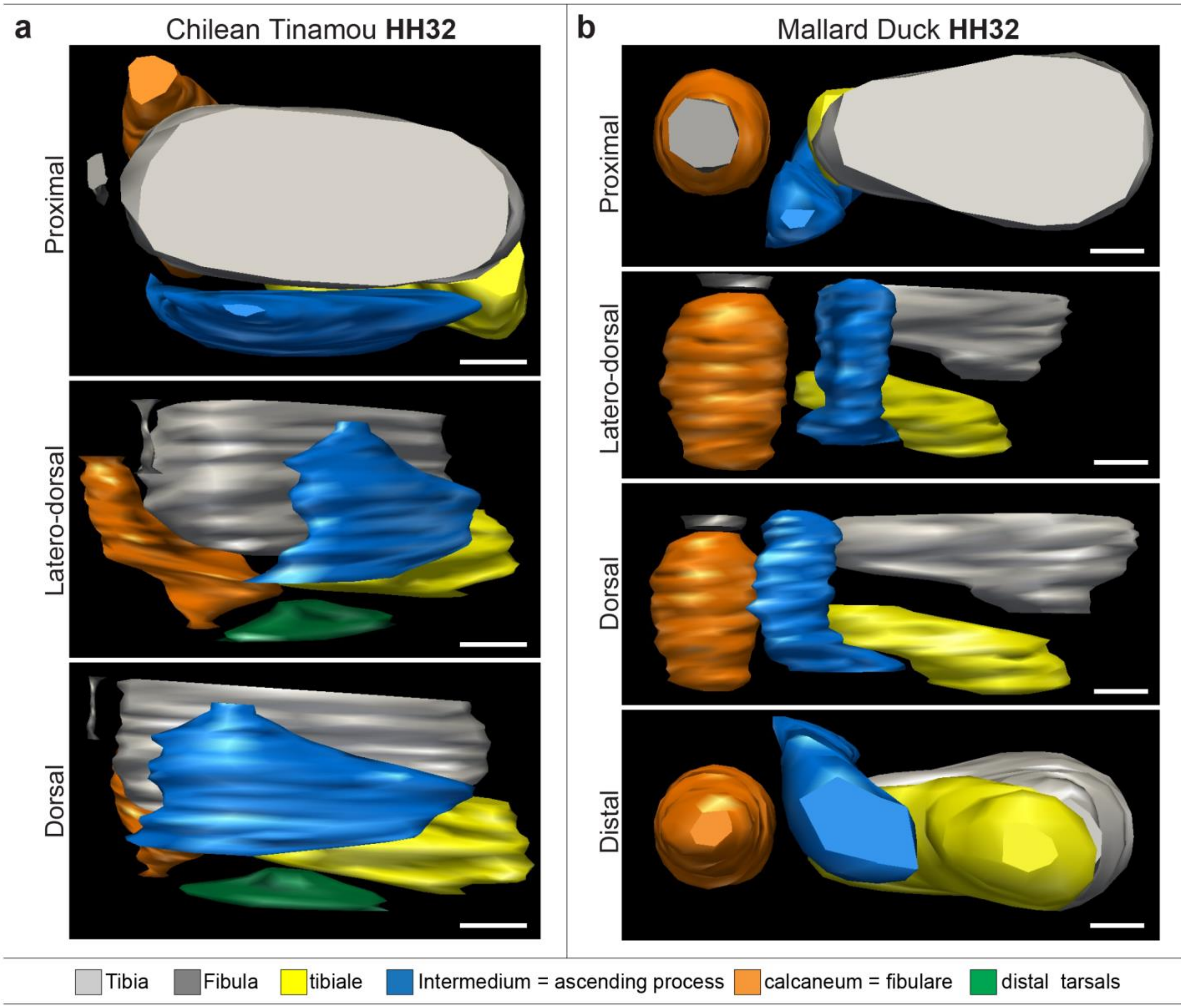

Supplementary Figure 9 | 3-D software reconstruction of embryonic bird ankles from stacks of histological sections (**a**) Stage HH32 of Mallard Duck and (**b**) Stage HH32 of Chilean Tinamou. 5 embryos per stage were used for Chilean tinamou and Mallard Duck. Scale bars=100 μm.

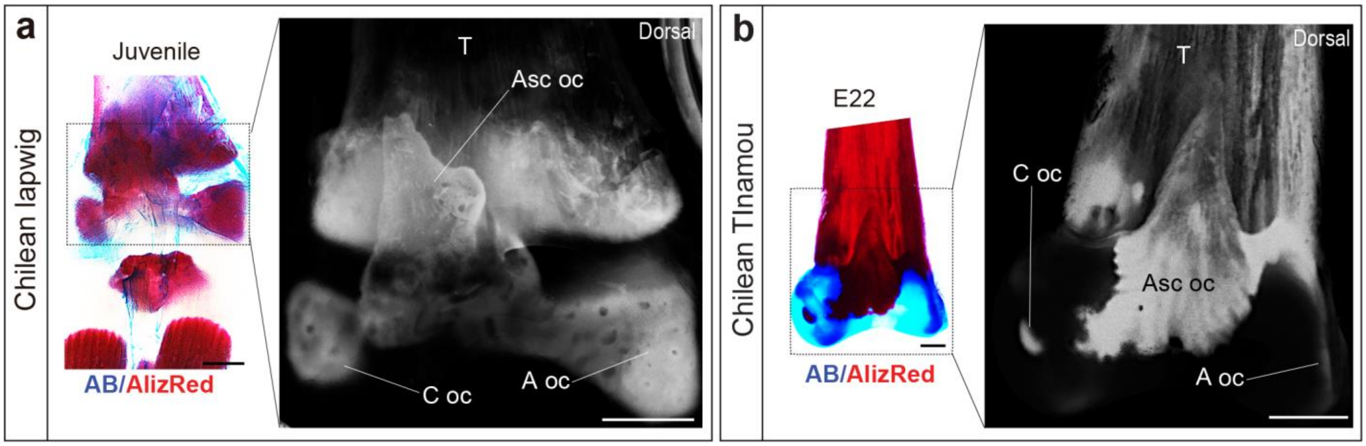

Supplementary Figure 10 | Variable position of the ASC in modern birds (**a**) In neognathous birds like the Chilean lapwing, the ossification of the ASC develops in a lateral position, contacting the calcaneum, and thus appears to project from this bone. (**b**) In paleognathous birds like the Chilean tinamou, the ASC is in a more central, dinosaur-like position, and appears to project from the astragalus. Our exhaustive embryological data clarifies that in modern birds, the ASC is not a projection of the astragalus or of the calcaneum, but an independent element: the intermedium, which comes into closer contact with the calcaneum (in neognaths) or the astragalus (in paleognaths). 2 embryos per stage were used for Chilean lapwing and Chilean tinamou. Abbreviations: T= Tibia C oc=Calcaneus ossification centre, A oc=Astragalus ossification centre, Asc oc=Ascending process ossification centre. Scale bars: (**a**, **b**) 1 mm.

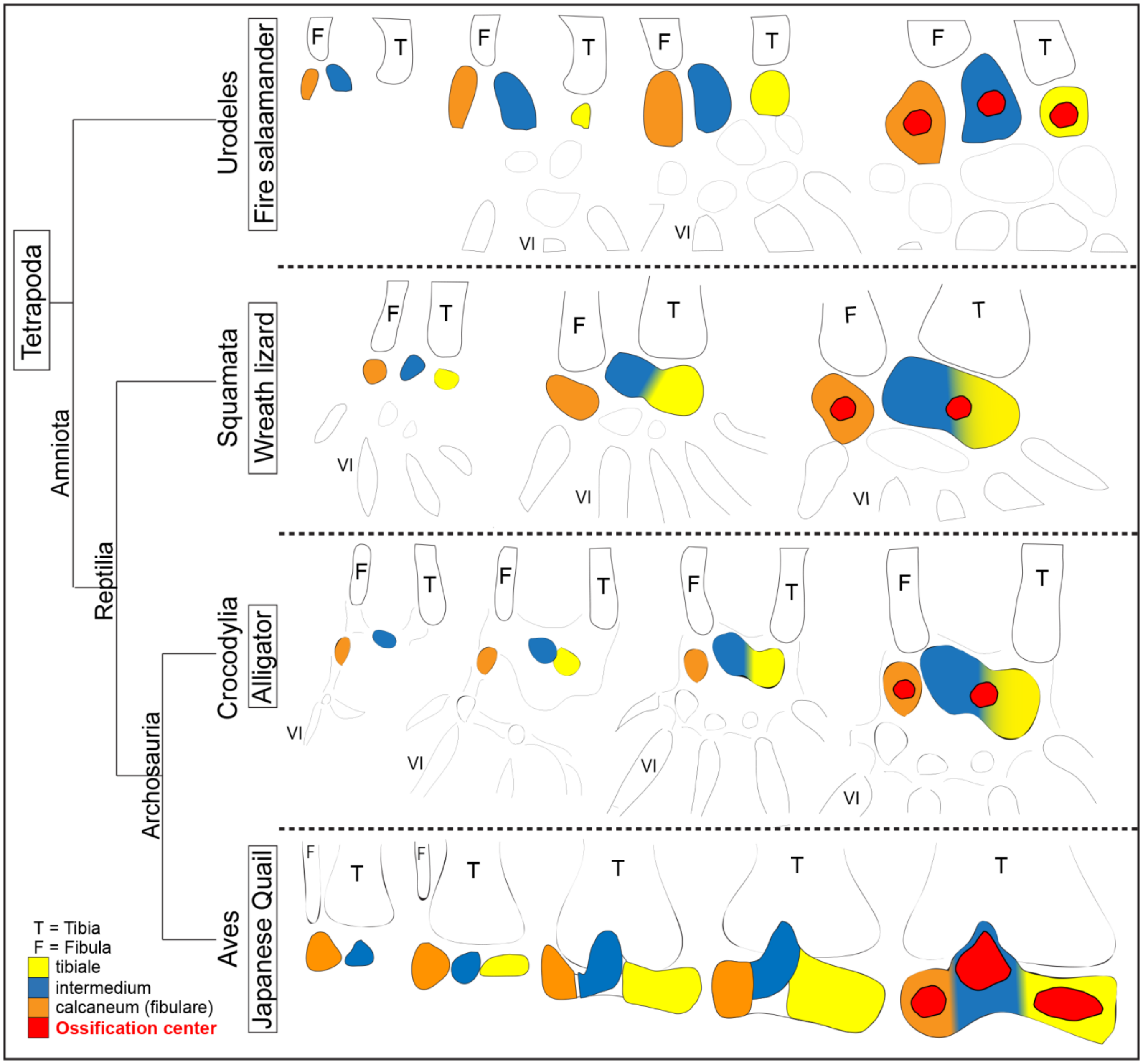

Supplementary Figure 11 | Evolutionary de-coupling of skeletal elements in the ankle of birds As in amphibians[3,5,6], but unlike other amniotes[1,2,3], the early intermedium cartilage of birds does not fuse early to the tibiale to form the astragalus. Only at a late stage, all the proximal cartilages (tibiale, intermedium and calcaneum) of birds fuse to form a large “distal cap” to the tibia. This cap then develops three ossification centres, at the position of each cartilaginous component. This includes an ossification of the intermedium whereas other amniotes only present two proximal ossifications, that of the astragalus and calcaneum. The fact the intermedium of birds does not fuse early and develops its own ossification centre suggests evolutionary reversion, resembling the condition once present in remote, amphibian-grade ancestors.

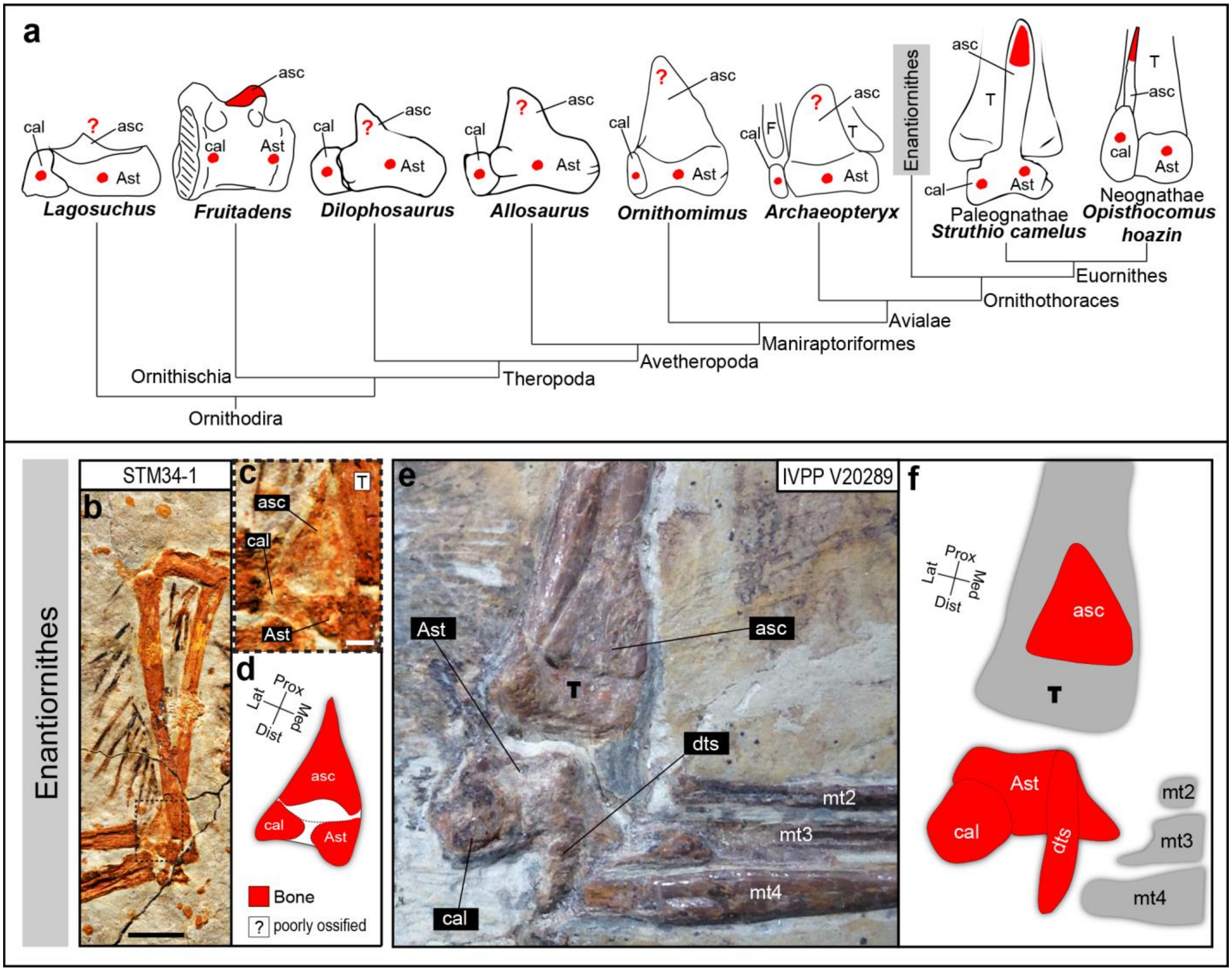

Supplementary Figure 12 | The evolutionary history of the ASC (**a**) The ASC originated as a small upward projection of the astragalus in early Ornithodira like *Lagosuchus.* A separate ossification has been reported in the ASC of the ornithischian *Fruitadens*, but this could be a convergence (homoplasy) with birds. In Theropod dinosaurs the ASC became larger and triangular, projecting onto the anterior-distal surface of the tibia. In *Archaeopteryx* and closely related theropods*,* the ASC is separated by a transverse groove from the main body of the astragalus, but it is unclear if this is evidence of a separate ossification[7, 8]. In modern birds like the ostrich (a paleognath) and the hoatzin (a neognath), the ASC develops a separate ossification centre. (**b**-**d**) The proximal ankle of STM34-1, an unnamed juvenile enantiornithine bird[9]. (**b**) Hindlimbs of STM34-1. (**c**) Zoom-in of the tarsal region indicated in (**b**) showing the left proximal tarsus in dorsal view: A triangular element shows ossified areas at the proximal, medial and lateral ends and an apparent absence of bone at the centre (also observable in the counterslab, data not shown) **(d)** Interpretative drawing of three ossifications within a cartilaginous “distal cap” of the tibia (**e**) Hindlimb of an unnamed enantiornithine specimen IVPPV20289. The ASC is separated from the astragalus and calcaneum. (**f**) Interpretative drawing showing the detached ossification of the ascending process. Abbreviations: T= tibia, cal=calcaneus, Ast=Astragalus, asc=ascending process, dts=distal tarsals, mt=metatarsals. Photographs in **b** and **c** were digitally flipped for easy comparison to other figures. Scale bars: (**b**) 5 mm, (**c**) 1 mm.

Supplementary References:

1. Müller, G. B. & Alberch, P. Ontogeny of the limb skeleton in Alligator mississippiensis: developmental invariance and change in the evolution of archosaur limbs. *Journal of Morphology*. **203**, 151–164 (1990).

2. Fabrezi, M., Abdala, V., Martínez M., Oliver, I. Developmental basis of limb homology in lizards. *Anatomical Record* **290**, 900–912 (2007).

3. Schaeffer, B. The Morphological and Functional Evolution of the Tarsus in Amphibians and Reptiles. *Bulletin of the American History Museum*. **78**, 396-472 (1941).

4. Burke, A. C. & Alberch, P. The development and homology of the chelonian carpus and tarsus. *Journal of Morphology* **186:** 119-131. (1985).

5. Alberch, P. & Gale, E. A., Size dependence during the development of the amphibian foot. Colchicine-induced digital loss and reduction. *Journal of embryology and experimental morphology* **76**,177-197 (1983).

6. Carroll, R. L. & Holmes, R. *Fins into Limbs.* (University of Chicago Press, Chicago and London, ed. 1, 2007) pp. 185-224.

7. Mayr, G., Pohl, B., Hartman, S., Peters, D. S. The tenth skeletal specimen of Archaeopteryx. *Zoological Journal of the Linnean Society* **149**, 97–116 (2007).

8. Chiappe, L. M., Shu'An, J., Qiang, J. Juvenile birds from the Early Cretaceous of China: implications for enantiornithine ontogeny. *American Museum Novitates*, **3594**, 1-46 (2007).

9. Zheng, X., Wang, X., O´Connor, J., Zhou, Z. Insight into the early evolution of the avian sternum from juvenile enantiornithines. *Nat. Commun*. **3**:1116 (2012).
